# Research progress of time-lapse imaging technology and embryonic development potential: A review

**DOI:** 10.1097/MD.0000000000035203

**Published:** 2023-09-22

**Authors:** JinLuan Wang, Ying Guo, Ning Zhang, TingTing Li

**Affiliations:** a School of Basic Medicine, Shanghai University of Traditional Chinese Medicine, Shanghai, China; b Reproductive and Genetic Center, Shandong University of Traditional Chinese Medicine Affiliated Hospital, Jinan, China; c Shandong University of Traditional Chinese Medicine, Jinan, China.

**Keywords:** dynamic parameters, embryonic development, time-lapse imaging

## Abstract

Cultivation and selection of high-quality human embryos are critical for the success of in vitro fertilization-embryo transfer. Time-lapse imaging technology (TLI) provides a stable culture environment for embryos, which can continuously observe and record the development process of early embryos, so that doctors can record embryo development time parameters more accurately. In this study, we review the current observation and research on the main embryo dynamics parameters in TLI and discusses their significance and development for embryo development potential. To analysis and summary, the application and research situation of TLI, we searched PubMed, Web of Science, and China National Knowledge Infrastructure, using TLI, embryo dynamics parameters, embryo development potential as Keywords, cited 50 out of the initial 89 selected literatures and summarized. With comparative analysis and research, we found that the embryo dynamic parameters provided by TLI has been intensively studied in clinical empirical and observational research, extensive experimental data verified its effectiveness and advantages in embryo development potential assessment. TLI provides technical support of embryo dynamic parameters, which may become the quantitative indicators for superior embryos and pregnancy prediction as well. Existing studies have shown that certain kinetic parameters provided by TLI culture can predict embryo implantation, but no parameter has been confirmed as the absolute correlation biological indicators yet. In this review we believe that further research is needed to verify these preliminary and sometimes contradictory results, and explore the predictive significance of various embryo kinetic parameters relying on TLI technology for embryo development potential.

## 1. Introduction

With the development of society, the change of people’s living environment and diet structure, the incidence of infertility has been on the rise in recent years. In the 1970s, the advent of assisted reproductive technology (ART)may bring hope for infertile patients. Due to the development of scientific and technological in more than 40 years, ART continues to innovate simultaneously. Using new technologies and methods to improve the clinical pregnancy rate and live birth rate of patients has always been the research goal and direction of reproductive doctors. In the process of in vitro fertilization-embryo transfer, the evaluation and selection of embryos are important factors that affect the pregnancy outcome of patients. The clinical pregnancy rate per cycle of traditional embryo selection methods is relatively low, about 30%.^[1]^ Many reproductive centers may choose to transfer multiple embryos at once to improve the success rate of embryo implantation for patients. For many patients with less embryos, especially elderly ones, multiple embryo transfer is more difficult, and increases the risk of multiple pregnancies, which is harmful to both the gravidas and fetuses. On the 1 hand, multiple pregnancies may increase the risk of a series of pregnancy-related diseases during pregnancy, such as diabetes and hypertension syndrome,^[2]^ and on the other hand, may increase the risk of fetal growth retardation, low weight, and organ dysplasia.^[3,4]^ Therefore, high-quality single embryo transfer is an effective method to achieve high pregnancy rates and avoid multiple pregnancies.

The selection of embryos mainly relies on the morphological evaluation, currently, by observing the number, size, and symmetry of nucleoli in the pronuclear stage of the embryo; the appearance and disappearance of pronuclei during cleavage, the number, symmetry, and fragmentation of blastomeres; morphological indicators such as the expansion and hatching status of the blastocyst, and the number of trophoblast cells can comprehensively predict the subsequent developmental potential of the embryo.^[5,6]^ Traditional morphological evaluation mainly relies on the technology and experience of embryologists, with a certain degree of inevitable subjectivity. Compared to embryos with low morphological scores, high score ones have a significantly higher probability of developing into high-quality blastocysts. However, in some studies, low-scoring embryos still have 25% chance of developing into high-quality embryos, with the same implantation potential as high-scoring embryos. Is not it a tragedy for these low-scoring embryos to be discarded miserably in clinical? ^[7–10]^ So time-lapse imaging (TLI) may become a breakthrough in rewriting this tragedy, by adding data quantificational perspective for embryo selection and playing a complementary role for the embryologist’s artificial scoring system, thus to make the prediction of embryo development potential more objective.

### 1.1. Main text

TLI introduces modern optical systems into traditional embryo culture modes, using time-lapse camera technology to expose and capture images of embryo development regularly, and provides a continuous and dynamic process of changes during embryo development more intuitively and objectively, avoiding the possibility of missing the appearance of 2 pronuclei in conventional culture, and increasing the utilization rate of eggs and embryos. It provides a stable external environment for embryo development and traceable data,^[11,12]^ enabling us to observe the early embryo development process and record embryo development time parameters more accurately, thus to predict the development potential of embryos, and helping us select a single high-quality embryo for transplantation effectively, to improve the final clinical pregnancy rate and live birth rate. This article reviews the current observation and research on the main embryo dynamics parameters in TLI and discusses their significance for embryo development potential.

## 2. Evaluate the kinetic parameters of the embryo

The value at which embryo development reaches a specific state or time point is called the embryo dynamics parameter. There is controversy over the names of specific embryonic dynamics parameter terms. Common terminology names include time, “cleavage cycle (cc)”, synchronicity, parameter, and interphase, wherein “time”, “cc,” “synchronicity” are used frequently. The initial reference for embryo division is t0, which is the time when the embryo undergoes fertilization. t0 is the time when sperm is injected into the oocyte in ICSI, but uncertain in IVF. Thus, Kaser and DJ^[13]^ advocates determining the time of the first cytokinesis groove (tcf1) as the standard reference for all cycles”.

The time when 2 blastomeres with independent cell membranes completely divide, and so on, t3, t4, and t16 (t2). Some studies have pointed out that during embryonic development, different developmental forms of embryos at a specific time point have certain predictive significance for embryonic development potential.^[14]^ Three previous studies showed that implantation embryos enter the 2-cell, 3-cell, 4-cell, 5-cell, and 8-cell stages of embryo development more quickly than non-implantation embryos, consistent with previous conventional morphological evaluation research results, indicating that embryos with faster cleavage rates have higher implantation potential.^[15–17]^

Different scholars have different definitions of the kinetic parameters represented by “cc”. Scholars such as Kirkegaard K^[17–19]^ use “cc” to indicate the time for doubling the number of cells, cc2 to indicate the time from 2-cell to 4-cell phase, and cc3 to indicate the time from 4-cell to 8-cell phase. Meseguer M^[20]^ and other scholars believe that “cc” refers to the duration of a certain cell phase, while cc2 refers to the duration of the embryo’s 2-cell phase, that is, the time interval of t3 to t2, and cc3 refers to the duration of the 4-cell phase, that is, the time interval of t5 to t4.Chamayou S^[21]^ define cc3 as the third cycle of cell division. The calculation method is to use the time of reaching the 5-cell phase to subtract the time of reaching the 3-cell phase. Their research reports that the optimal cc3 range is 9.7 to 21 hours, during which the implantation rate is the highest.

“S2” represents the duration of the embryo at the 3-cell stage, that is, the time interval of t4 to t3.^[17,20,21]^ Chamayou S^[21]^ and other scholars believe that s3 represents the duration from 5-cell to 8-cell transformation, that is, the time interval between t8 to t5. FreourT^[16]^ defined s3 as the time from the 4-cell stage to the 8-cell stage of an embryo. Different measurements or definitions of TLI parameters have a significant impact on the final research results and may even lead to conflicting results. Forecourts have found that the s3 parameter of embryonic development, namely, the duration of cleavage from 4-cell to 8-cell stages, can predict pregnancy (odds ratio = 2.8, 95% confidence interval 1.2–6.6). However, studies by Chamayou S^[21]^ and Kirkegaard K^[18]^ have not demonstrated a difference in the synchronization of the third cleavage division (s3, i.e., from 5-cell to 8-cell stages) between implanted and non-implanted embryos. It has been reported in the literature that t4 and s2 are different between euploid and aneuploid embryos.^[22]^

## 3. Study on dynamic parameters of TLI culture at different stages of embryo development

### 3.1. Evaluation of embryo dynamics from prokaryotic to cleavage stages

A series of changes occur in the oocyte after fertilization, such as the appearance and disappearance of the pronucleus, the appearance of the first cleavage, and so on. The period from prokaryotic stage to cleavage stage is an important period of early embryo development, during which embryo dynamics parameters, cleavage mode, and synchronization of cleavage have important reference significance for the normal development of embryos.^[23]^

Several studies have demonstrated that the early division of embryos on day 1 and the total number of cells on day 3 have important clinical significance in predicting the implantation potential of embryos.^[24–27]^ Most reproductive centers evaluate and score the development of embryos on day3 after fertilization. TLI provides continuous photo observation including the first, second, and third cleavage of early embryo division. Embryologists can predict subsequent blastocyst formation and embryo implantation from those key embryo dynamics parameters. Wong CC et al^[28]^ found that the duration of the first cytokinesis and the duration of nuclear reappearance after the first cytokinesis can predict blastocyst development. Subsequently, somebody retrospectively compared the morphological and dynamic characteristics of implanted and non-implanted embryos and found that there was a significant difference in the average duration of the 2-cell stage (11.8 + 1.2 vs 11.8 + 3.3 hours, *P* < .05) and 3-cell stage (0.78 + 0.73 vs 1.77 + 2.83 hours, *P* < .05) between the 2 groups, However, it is not possible to predict the final pregnancy outcome. Studies by scholars such as Chang Hui^[29]^ have found that, compared with normal women, patients with polycystic ovary syndrome have longer t2, t3, t7, and t8, and significantly lower embryo implantation rates and pregnancy rates, suggesting that t2, t3, t7, and t8 have certain predictive significance for the clinical pregnancy outcomes of polycystic ovary syndrome patients. Recent studies suggest that blastocysts with rapid development are more likely to be euploid embryos.^[30–32]^In 2020, Zou Yaoyu et al^[30]^ conducted a retrospective analysis of 550 embryos from 150 preimplantation genetic testing assisted pregnancy patients. It was found that the t4 of aneuploid embryos was significantly longer than euploid embryos, and the S2 distribution shifted to the right, while tHB had an increasing trend. Another study found that, except for t8, the division time point during the entire process of development of euploid embryos from tPNf to tB was earlier than that of aneuploid embryos.^[31]^ Therefore, embryo dynamics assessment based on TLI from the prokaryotic stage to the cleavage stage, can provide scientific and objective data for the selection of embryos in the laboratory and for clinical doctors, to cross reference with laboratory manual selection and make final decisions.

### 3.2. Evaluation of embryo dynamics during blastocyst stage

In the in vitro fertilization-embryo transfer process, blastocyst transfers are the preferred option for many patients because of closing to the embryo situation in a natural pregnancy state, and more fit for human physiology. Conventional morphological evaluation cannot accurately predict the specific situation of embryo development due to the large differences in embryonic development. Traditional embryo screening may miss potentially high-quality embryos and discard them, or choose abnormal fertilized embryos to transfer, which to some extent reduces the clinical pregnancy rate. Based on the above reasons, some reproductive centers tend to conduct blastocyst transplantation in order to improve pregnancy outcomes. The key to blastocyst formation is the emergence of the blastocyst cavity. The blastocyst ultimately forms completely when the blastocyst cavity fills the entire embryo. In 2013, it was reported that the time of blastocyst formation and development can predict embryo implantation.^[33]^In a subsequent study, the authors retrospectively applied the aneuploid risk model to implantation, using morpho-dynamic analysis and trophoblastic ectoderm biopsy to determine euploid/aneuploid pairing. Embryos were divided into low, medium, and high aneuploid risk groups based on the duration of blastocyst development. The results showed that in the low aneuploid risk group, the blastocyst development took 96.2 hours and was fully developed; in the middle aneuploid risk group, the blastocyst developed ≥ 96.2 hours, and developed completely; while the blastocyst development delay in the high aneuploid risk group was ≥ 122.9 hours. As the risk of embryo aneuploidy increases, the embryo implantation rate gradually decreases (low: medium: high = 72.7%: 25.5%: 0%).^[34]^ Thus, it is possible to avoid selecting embryos with a high risk of aneuploidy during blastocyst transfer combining embryo dynamics parameters to improve pregnancy outcomes.

TLI has been proven to improve blastocyst prediction outcomes and reduce both inter-and intra-observer variability, compared to traditional morphological assessments.^[35–37]^ In fact, reliable prediction of blastocyst transformation based on early morpho-dynamic markers may be a major advantage of TLI. In 2013, Conaghan J et al^[36]^ conducted a prospective study on this issue, selecting 3 embryologists to form an observation group to predict the development ability of blastocysts. They conducted research based on traditional day 3 morphology and TLI plus day 3 morphology, respectively. The results showed that the addition of TLI decreased inter-observer specificity, while significantly improving the specificity of accurately predicting blastocyst development (52.1% vs 84.7%, *P* < .05).

## 4. Advantages and security of TLI

Traditionally, embryo morphological evaluation is performed by removing embryos from a conventional incubator daily and observing them under a microscope by an embryologist to assess their quality. This approach exposes embryos to potentially suboptimal environments outside of incubators and human processing, with certain potential adverse effects on embryo development. TLI culture places a camera in the incubator, captures digital images of embryos at set time intervals (for example, every 5 to 15 minutes), and forms continuous images through software editing to create a delayed sequence of embryo development, eliminating the need to repeatedly take out embryos for observation, thereby limiting the exposure of embryos to changes in gas composition, external temperature, and motion, creating a stable environment for embryo growth and development, reduce the impact of external environment and human operations on embryo development. TLI provides important information’s, including the timing of cell division, the intervals between cell cycles, and other developmental conditions (such as dynamic prokaryotic patterns, the presence of multinucleation and division, and blastomere symmetry). Accumulates detail delayed images of embryonic development at fixed time intervals. According to the literature, abnormal dynamic cleavage patterns during embryo development can significantly affect the implantation rate of embryos, and embryos with abnormal cleavage have a higher abortion rate after transplantation.^[38]^ The time interval set for conventional embryo morphological evaluation is long, and many characteristics of transient events may be missed. TLI has been used by multiple centers at home and abroad to show higher survival rates in culture,^[39,40]^ reducing the early pregnancy abortion rate.^[41]^ The effect is superior to traditional embryo culture systems.^[41,42]^Zhao Xiaoli et al^[43]^ found that the application of TLI can improve the success rate of first transfer in patients with high-frequency polyspermy fertilization. Li Guozhen et al^[44]^ found that the TLI system can significantly improve oocyte utilization. The TLI optimization model is a simple embryo optimization scheme; when the number of retrieved eggs > 10 or the number of D3 available embryos/transferred embryos > 3, the implantation rate and clinical pregnancy rate of the TLI embryo preference scheme may be improved compared to the conventional preference scheme.

Due to regular image collection of embryos in TLI culture, the risk of regular exposure of embryos is increased. Some studies have found that embryos exposed to short wavelength light for a long time have a higher probability of developing abnormalities.^[45]^ Chen Yuyang^[46]^ compared the normal fertilization rate and embryo implantation rate of embryos in TLI incubator with those in the traditional incubator, and no significant abnormality was found. A retrospective cohort study by Reignier A et al^[47]^ found that among couples receiving ICSI (Intracytoplasmic sperm injection) treatment, the total cumulative live birth rate in the TLI group was significantly higher than in the conventional incubation group (66.9% vs 56.4%, *P* = .02). Therefore, the impact of periodic exposure to TLI on embryo development remains to be discussed and needs to be demonstrated through more research. TLI is a new method of embryo observation, and its specific impact on embryo development needs to be further explored. We also need to continue to pay attention to and evaluate the safety of TLI culture systems.

## 5. Artificial intelligence development of TLI

TLI enables embryologists to obtain standardized embryo pictures with identical definitions, unlike manual evaluation with microscopes. In the past few years, artificial intelligence has been applied to ART, allowing embryologists to manage hundreds of TLI digitalized images of embryo development. Denmark scholar Jørgen Berntsen et.al,^[48]^ build a fully automated iDAScore v1.0 model, which was trained and evaluated based on a large dataset from 18 IVF centers consisting of 115,832 embryos, of which 14,644 embryos were transferred KID embryos model predictions correlated positively with blastocyst grading and negatively with direct cleavages. This deep learning-based embryo selection model using only time-lapse image sequences performed across different patient ages and clinical conditions, which was shown to perform at least as good as a state-of-the-art manual embryo selection model. Full automatization of embryo scoring implies fewer manual evaluations and eliminates biases due to inter- and intra-observer variation. This model provides an excellent example and direction for the further development of TLI.

TLI provides novel morpho-kinetic and morphometric parameters, supports the selection of a single embryo for transfer, allows embryologists to identify unknown or undetectable features of embryo development, including atypical phenotypes such as direct or reverse cleavage and blastocyst collapse(s) episode, and help embryologists to accurately master the embryonic development to obtain a healthy pregnancy to term. Sciorio R et.al^[49]^ believe that presumably, in the future, with the further advancement of time-lapes monitoring and artificial intelligence, clinical embryologists will move to an established, full automated and noninvasive analytical method for embryo selection, ensuring an elevated pregnancy outcome.

## 6. Summary and outlook

Embryo evaluation and selection are based on the comprehensive performance of the entire in vitro fertilization process. Selecting the best single embryo for transfer is the basic goal of all embryologists. The emergence of TLI provides technical support for better embryo selection and pregnancy prediction, introducing an increasing amount of research on embryonic development parameters in clinical reproductive medicine, a large amount of data was accumulated for further research. We reviewed the latest research progress of TLI and the embryonic development parameters, provided new reference research directions of TLI for researchers hoping to bring new assistance to the prediction of embryo development potential. Although existing studies have shown that certain kinetic parameters during TLI culture can predict embryo implantation, there is no single kinetic parameter that can consistently predict embryo development potential. Further research is needed to verify these preliminary and sometimes contradictory results, and explore the predictive significance of various embryo kinetic parameters relying on TLI technology for embryo development potential, with a view to improving the pregnancy outcome of patients.

## Acknowledgements

This research was fully sponsored by National Natural Science Foundation of China with grant number 81804130 (Research of the kidney deficiency’s essence and reproductive function decline after the age of “Five-Seven” in Huang Di Nei Jing based on mitochondrial energy metabolism pathway)

## Author contributions

**Funding acquisition:** Ying Guo.

**Methodology:** Ying Guo.

**Supervision:** Ning Zhang.

**Writing – original draft:** JinLuan Wang.

**Writing – review & editing:** TingTing Li.
